# Characterization of genetic heterogeneity within accessions in the USDA soybean germplasm collection

**DOI:** 10.1002/tpg2.20000

**Published:** 2020-04-06

**Authors:** Nicole T. Mihelich, Steven E. Mulkey, Adrian O. Stec, Robert M. Stupar

**Affiliations:** ^1^ Department of Agronomy and Plant Genetics University of Minnesota Saint Paul MN 55108 USA

## Abstract

Soybean breeding relies on the use of wild (*Glycine soja* Sieb. and Zucc.) and domesticated [*Glycine max* (L.) Merr.] germplasm for trait improvement. Soybeans are self‐pollinating and accessions can be maintained as pure lines, however within‐accession genetic variation has been observed in previous studies of some landraces and elite cultivars. The objective of this study was to characterize within‐line variation in the accessions housed in the USDA Soybean Germplasm Collection. This collection includes over 20,000 accessions, each previously genotyped using the SoySNP50K Chip. Each SoySNP50K genotype was developed by pooling approximately three individuals per accession. Therefore, clusters of SNPs called as heterozygous within an accession can be inferred to represent putative regions of heterogeneity between the three individuals sampled. In this study, we found high‐probability intervals of heterogeneity in 4% of the collection, representing 870 accessions. Heterogeneous loci were found on every chromosome and, collectively, covered 98.4% of the soybean genome and 99% of the gene models. Sanger sequencing confirmed regions of genomic heterogeneity among a subset of ten accessions. This dataset provides useful information and considerations for users of crop germplasm seed banks. Furthermore, the heterogeneous accessions and/or loci represent a unique genetic resource that is immediately available for forward and reverse genetics studies.

**Abbreviations**
GRIN: Germplasm Resources Information Network
HIF: heterogeneous inbred family
NIL: near isogenic line
PCR: polymerase chain reaction
RIL: recombinant inbred line
SGC: Soybean Germplasm Collection
SNP: single nucleotide polymorphism
UMGC: University of Minnesota Genomics Center

## 1 INTRODUCTION

Soybean is a globally important food and feed crop grown on over 120 million hectares around the world in 2017 (FAOSTAT Database, 2019). The genetic diversity of cultivated soybean is narrow due to the limited subset of wild progenitors from which it was domesticated, along with the intense selection of modern breeding. Moreover, soybean is a self‐pollinated crop with relatively low rates of outcrossing (Ray, Kilen, Abel, & Paris, 2003). While it is often assumed that elite cultivars are highly homogeneous, within‐accession variation has continued to be observed in elite germplasm (Fasoula & Boerma, 2005; Haun et al., 2011). During the breeding process, this originates from the segregation and fixation of residual heterozygous loci following single seed descent. Successive bulk harvesting in later generations maintains the heterogeneity among individuals of the cultivar. This within‐accession heterogeneity has been successfully exploited in soybean breeding to select for superior individuals within established cultivars, resulting in the development of new lines (Fasoula & Boerma, 2007; Sebastian et al., 2010).

Soybean within‐accession variation has been characterized at the molecular level in the reference cultivar ‘Williams 82’ (Haun et al., 2011). The original development of ‘Williams 82’ into a cultivar involved pooling four lines from an BC_6_F_3_ introgression of a Phytopthora root rot resistance locus from the accession ‘Kingwa’ into the elite cultivar ‘Williams’ (Bernard & Cremeens, 1988). Theoretically, the BC_6_F_3_ plants would have over 98% of their genome from ‘Williams’ leaving approximately 2% as ‘Kingwa’ to differentially segregate among the four pooled individuals. Within‐accession heterogeneity was found among independently maintained stocks of ‘Williams 82’ (Haun et al., 2011). A major region of within‐accession heterogeneity was found in the Phytopthora root rot resistance introgression site on chromosome 3, along with several other notable intervals on chromosomes 7, 14, 15, and 20. In this sense, each ‘Williams 82’ sub‐line acts as a near‐isogenic line (NIL) to the other ‘Williams 82’ sub‐lines (Haun et al., 2011).

The insights on within‐accession heterogeneity from ‘Williams 82’ draws attention to its potential widespread prevalence within other soybean germplasm. In addition to breeding‐driven forces, within‐accession heterogeneity could result from natural genomic alterations (e.g., mutation, illegitimate recombination), or disruption of typical mating systems (e.g., outcrossing in an otherwise self‐pollinating species). Therefore, one might expect to find genetic heterogeneity not just within cultivars, but also within accessions of different germplasm sources, ranging from modern cultivars to wild relatives.

Within‐accession heterogeneity has largely been uncharacterized in soybean. The United States Department of Agriculture (USDA) Soybean Germplasm Collection (SGC) contains 20,087 wild and cultivated soybean accessions that were each recently genotyped with over 42,000 single nucleotide polymorphism (SNP) markers using the SoySNP50K Illumina Chip (Song et al., 2013; Song et al., 2015). The DNA samples used to generate the SoySNP50K dataset were derived from pooling multiple (approximately three) individuals from each accession (Dr. David Hyten, personal communication). Theoretically, genomic regions that are heterogeneous between the pooled individuals in a given accession would be detected as heterozygous SNP calls on the SoySNP50K Chip (see Supplemental Figure S1).

In this study, we sought to identify potential heterogeneous genomic intervals in each of the 20,087 USDA SGC accessions using the SoySNP50K, as shown in Supplemental Figure S1. Germplasm‐wide detection of these intervals is valuable for at least two reasons: (1) Users of seed bank resources should be aware of deviations from genetic uniformity of accessions both within and among collections; (2) Accessions with heterogeneity in a genomic region of interest could be used to immediately identify sets of NILs, enabling trait mapping, gene validation, uncovering untapped variation, and advancing breeding efforts.

**Core Ideas**

- Over 20,000 soybean accessions were screened for within‐line heterogeneity
- Heterogeneous loci were found in 870 accessions, spanning 99% of the gene models
- Heterogeneous accessions can be used to rapidly identify near‐isogenic lines

## 2 MATERIALS AND METHODS

### 2.1 USDA soybean germplasm collection data procurement

The dataset that was used for this analysis was the cultivar ‘Williams 82’ assembly 2 (Wm82.a2) SoySNP50K VCF data (Song et al., 2013; Song et al., 2015) downloaded from SoyBase (http://soybase.org) (Grant, Nelson, Cannon, & Shoemaker, 2010) on 5 Sept. 2017. At the date of download, this dataset featured the 20,087 *G. max* and *G. soja* accessions from the USDA SGC. As described above, the DNA samples used in this dataset were derived from pooling a small number of individuals from each accession. Therefore, genomic intervals of heterogeneity within accessions were inferred from heterozygous SNP calls on the SoySNP50K Chip (Supplemental Figure S1). It can be assumed that the majority of the heterozygous calls are a mixture of different homozygous calls rather than heterozygosity within individuals, as soybean is a self‐pollinating and highly inbred species.

### 2.2 Heterogeneous accession screening

For each USDA SGC SoySNP50K accession, the number of heterozygous calls on each chromosome were counted, including the scaffolds as one group, using R 3.4.3 in RStudio 1.1.414. Only two SNP markers were from the mitochondrial genome and were thus insufficient to be included in analysis. The three chromosomes on each genotype with the highest SNP counts were determined. A pipeline was developed to identify the accessions that had discrete regions of potential heterogeneity, while filtering out accessions with exceedingly high rates of heterozygous calls, as those may indicate poor genotyping and/or contamination of the DNA sample. There were three different ways for an accession to be identified as having regions of heterogeneity in the genome, identified in succession: (1) Accessions with at least one major chromosome with heterogeneity (named as the “One Chromosome” class) were identified when the chromosome with the highest number of heterozygous SNPs contained greater than 50% of the total heterozygous SNPs in the sample, and the total number of heterozygous calls in the sample was greater than 10. (2) Accessions that were not previously identified by the criteria of the “One Chromosome” class, but had at least two major chromosomes with heterogeneity (named as the “Two Chromosomes” class) were identified when the chromosome with the second highest number of heterozygous SNPs contained greater than 25% of the total SNPs in the sample, and the total number of heterozygous calls in the sample was greater than 20. (3) Accessions that were not previously identified with the criteria of the “One Chromosome” or “Two Chromosomes” classes, but had at least three major chromosomes with heterogeneity (named as the “Three Chromosomes” class) were identified when the chromosome with the third highest number of heterozygous SNPs contained greater than 15% of the total SNPs in the sample, and the total number of heterozygous calls in the sample was greater than 30. Any combination of these three criteria could result in a potential interval or intervals of heterogeneity. While these categories were designed to find one, two, or three chromosomes with clear heterogeneity, it was expected that more chromosomes would contain intervals of heterogeneity beyond this initial screen. These parameters were chosen to distinguish patterns of heterogeneity from heterozygous calls caused by genotype contamination, which would likely appear more evenly distributed throughout the genome. These parameters were also chosen to capture different patterns of heterogeneity, from one large major region on one chromosome, to several small regions on different chromosomes (Supplemental Figure S2).

### 2.3 Heterogeneous interval detection

Once a subset of accessions was identified with the screening criteria, intervals of heterogeneity were detected with R 3.4.3 in RStudio 1.1.414. No concentrated heterogeneity was detected in any scaffolds, and thus were not included for interval detection. First, a matrix resulting from a sliding window of 21 adjacent SNPs was created to coarsely detect clusters of heterozygous calls throughout the 42,080 SNPs of the genome for each accession. Intervals were called heterogeneous if there were at least three heterozygous SNPs within a 21 SNP sliding window on a given chromosome. Once an interval was detected, the borders were defined by the first and last heterozygous SNP calls. The sliding window was adjusted at the extreme ends of the SoySNP50K data (beginning of chromosome 01 and the end of chromosome 20) to ensure inclusion of all SNPs during detection. Since the SoySNP50K SNP call file is formatted continuously and consecutively from one chromosome to the next, the analysis also accounted for chromosomal boundaries to prevent spurious intervals that could span across different chromosomes. The overall mean and median interval size were determined on both a SNP and physical genomic size basis. The interval size in SNPs was counted as the number of SNPs between and including the first and last heterozygous SNPs, and counted all SNP types: heterozygous, homozygous (reference and alternate), and missing SNPs within the SoySNP50K SNPs. The interval size in kilobases was counted similarly, using the difference in the physical positions of the first and last heterozygous SNPs. Proportions of detected heterogeneity were then compared in *G. soja*, landrace, North American cultivars, and unclassified *G. max* accessions. Landraces and North American cultivar classes were defined by Song et al. (2015). Unclassified *G. max* accessions comprise a combination of landraces, North American cultivars, non‐North American cultivars, and other un‐released breeding materials that were not previously defined in Song et al. (2015).

Coverage calculations were performed using modules on the Minnesota Supercomputing Institute (MSI). Base pair coverage calculations were performed using bedtools 2.25.0 (Quinlan & Hall, 2010) genomecov, with a BED file of the chromosome, start, and stop position of all detected heterogeneous intervals, as well as a TXT file of the size of each soybean chromosome in bases from SoyBase (http://soybase.org) (Grant et al., 2010). Base pair coverage calculations concerning euchromatic and heterochromatic regions were defined from Song et al. (2016) and calculated in the same way as the overall genome coverage after isolating chromatin types with bedtools 2.25.0 intersect of the Song et al. (2016) boundaries. Gene model coverage calculations were performed using bedtools 2.25.0 intersect, with the GFF file of all genes in the reference genome Wm82.a2.v1 (downloaded from SoyBase 17 Dec. 2014) intersected with a BED file of the chromosome, start, and stop position of all detected heterogeneous intervals. The option ‐f 1.0 was used to ensure heterogeneous intervals overlapped with the entire gene model sequence.

### 2.4 Validation of heterogeneity

To confirm that our pipeline was detecting true heterogeneity, ten accessions each exhibiting one region of putative heterogeneity (members of the “One Chromosome” class) were selected for validation. Four of these (PI 79712, PI 423780B, PI 542043, PI 595926) are landraces or cultivars maintained by the USDA SGC under standard conditions. Six accessions have known male sterile or lethal chlorosis loci and are maintained by the USDA SGC as heterozygotes according the USDA Germplasm Resources Information Network (GRIN).

Seed samples for the accessions were obtained from the USDA SGC in March 2019. DNA was extracted from seed chips using BioSprint 96 DNA Plant Kits (QIAGEN) following the manufacturer's protocols. DNA was extracted from twelve separate seeds for each accession. For each of the ten accessions, PCR primers were designed to amplify a single SNP within the detected heterogeneous interval. Specific SNPs were chosen based on a central location within the heterogeneous interval and a heterozygous genotype call in the SoySNP50K Chip data. Primer design was performed using Primer3Plus (Untergasser et al., 2007). Amplified PCR products were genotyped at the University of Minnesota Genomics Center (UMGC) core facility using Sanger sequencing.

## 3 RESULTS

### 3.1 Heterogeneous accession scan and intervals detected in the USDA SGC

The USDA SGC was previously genotyped with 42,080 markers across 20,087 accessions (Song et al., 2015). For each accession, the DNA was a pooled sample of approximately three individuals. As soybean is a self‐pollinating species, the vast majority of SNP calls were homozygous. However, genomic regions with clusters of heterozygous calls were also observed in some accessions, suggesting the presence of either remnant heterozygosity in some individuals or sequence heterogeneity among the pooled individuals (Supplemental Figure S1). Given the inbred nature of soybean, pureline maintenance of the USDA SGC, and the pooling of samples during genotyping, heterozygous calls were inferred to primarily represent heterogeneous bases that were homozygous within each respective individual (Supplemental Figure S1).

We were interested in identifying those accessions that exhibited heterogeneity, characterizing the frequency and extent of heterogeneity within accessions, and validating the heterogeneity at a single locus among a subset of individuals. The SNP genotype profile of each accession was evaluated in a two‐stage analysis pipeline to first identify highly heterogeneous accessions and second describe their heterogeneous intervals. The primary analysis screened for heterogeneous accessions by identifying individual chromosomes that had disproportionately high heterozygous calls compared to the overall genomic distribution of heterozygous calls. To detect accessions with true heterogeneous intervals while filtering out accessions that had few or unreasonably frequent heterozygous calls dispersed throughout the genome, the pipeline was designed to identify accessions that had either one, two, or three chromosomes with disproportionately high heterozygous call rates (Supplemental Figure S2). Of the 20,087 accessions in the USDA SGC, 885 (4.4%) passed the accession screening parameters (see Materials and Methods for details about the screening parameters). Of those detected, 448 genotypes had only one heterogeneous chromosome detected (“One Chromosome” class), 187 genotypes had two heterogeneous chromosomes detected (“Two Chromsomes” class), and 250 had three heterogeneous chromosomes detected (“Three Chromosomes” class; Table 1).

**TABLE 1:** Results of the heterogeneity analysis pipeline for 20,087 *G. max* and *G. soja* accessions from the USDA Soybean Germplasm Collection, based on initial screening parameters that assigned accessions to one of three categories based on the distribution of heterogeneity. The number of accessions which passed initial screening are presented, as well as accessions that passed the secondary interval detection step. Also presented are the mean and median number of heterogeneous intervals for each category of accessions

Heterogeneity screening category^a^	No. of accessions	No. of accessions with intervals detected^c^	Mean no. intervals per accession	Median no. intervals per accession
One Chromosome^b^	448	442	4.3	3
Two Chromosomes	187	187	8.3	6
Three Chromosomes	250	241	14.3	12
Total	885	870	8	5

a Accessions were assigned to one of three categories based on the distribution of heterozygous SNP calls using the Soy50KSNP Chip data.

b “One Chromosome”: at least 10 heterozygous SNP calls, >50% of which were located on one chromosome; “Two Chromosomes”: not previously identified by the criteria of the “One Chromosome” class, at least 20 heterozygous SNP calls, >25% of which occurred on the second most ‘heterogeneous’ chromosome; “Three Chromosomes”: not previously identified with the criteria of the “One Chromosome” or “Two Chromosomes” classes, at least 30 heterozygous SNP calls, >15% of which occurred on the third most ‘heterogeneous’ chromosome.

c A heterogeneous interval was called when at least 3 heterozygous SNPs were observed in a 21 SNP sliding window.

The 885 accessions identified by screening were then analyzed to determine the heterogeneous intervals across their genomes. Intervals were detected by identifying clusters of at least three heterozygous SNPs within a sliding window of 21 SNPs. Interval borders were defined by the first and last heterozygous SNP calls that were detected before the sliding window reached zero heterozygous calls. After interval detection, 870 of the 885 accessions were confirmed to have at least one heterogeneous interval (Table 1). The 15 (1.7%) accessions from the primary screening that did not have heterogeneous intervals detected were close to the minimum cutoffs of the primary screening, and instead of forming clusters, the heterozygous SNPs were dispersed throughout the chromosome(s). These findings demonstrated that the accession screening was an effective initial identification of accessions with heterogeneity. The pipeline was designed to filter out most poorly‐genotyped or contaminated samples (e.g., Supplemental Figure S2B), and resulted in a low number of false positive identifications.

Among the 870 positive accessions, a total of 6,925 putatively heterogeneous intervals were detected (Supplemental Table S1). The mean and median number of intervals detected from the second stage of analysis were several times greater than the number of chromosomes found in the accession screening categories (Table 1). There were two reasons for this. First, the accession screening was designed to specifically search for outlier chromosomes. However, when scrutinized more closely with the heterogeneous interval detection step, smaller intervals on other chromosomes were also able to be detected. Second, the chromosomes detected in the accession screening often contained multiple intervals. These were only reported as a single chromosome in the accession screening but were resolved as multiple and distinct intervals in the sliding window analysis.

**FIGURE 1:**
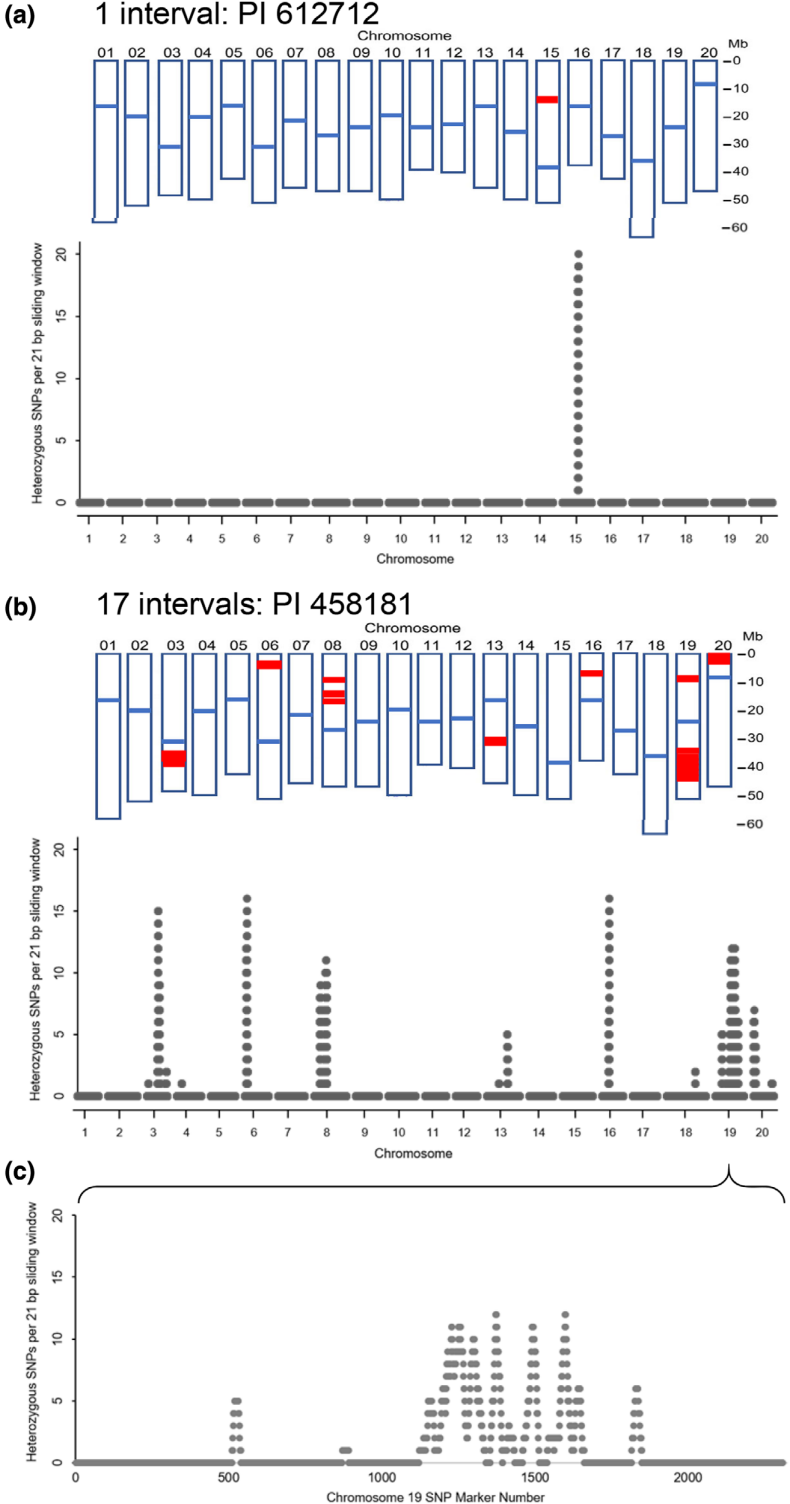
Examples of accessions with detected intervals of heterogeneity. Part (a) represents PI 612712, an accession of unclassified *G. max* with one chromosome detected in heterogeneous accession screening as well as one interval detected on the chromosome in heterogeneous interval detection. Part (b) represents PI 458181, a landrace with three chromosomes detected in heterogeneous accession screening and seventeen intervals detected on seven different chromosomes in heterogeneous interval detection. Part (c) represents chromosome 19 of PI 458181, which was magnified to show the seven intervals detected on one chromosome. White bars represent each chromosome of the soybean genome with approximate length in megabases (Mb). Blue bars on each chromosome represent the approximate centromeric region. Red bars indicate occurrences or clusters of heterozygous SoySNP50K calls (Song et al., 2015) within a chromosome. Gray dots represent 21‐SNP sliding windows throughout the 42,080 SNPs of the genome used to coarsely detect clusters of heterozygous calls for each accession

Different types of heterogeneous patterns were observed in this study (Figure 1). On one end of the extreme, *G. max* PI 612712 had a single, clear 412 kb (60 SNPs) interval on chromosome 15 (Figure 1a). In contrast, *G. max* PI 458181 had 17 intervals among seven different chromosomes (Figure 1b). Accessions such as this, with many intervals detected, often showed both a wider distribution of intervals throughout the chromosomes and more intervals clustered within a chromosome, such as the 7 intervals on chromosome 19 (Figure 1c). However, when multiple intervals were detected on a chromosome, they were not always clustered.

### 3.2 Heterogeneous intervals detected by soybean germplasm classes

**FIGURE 2:**
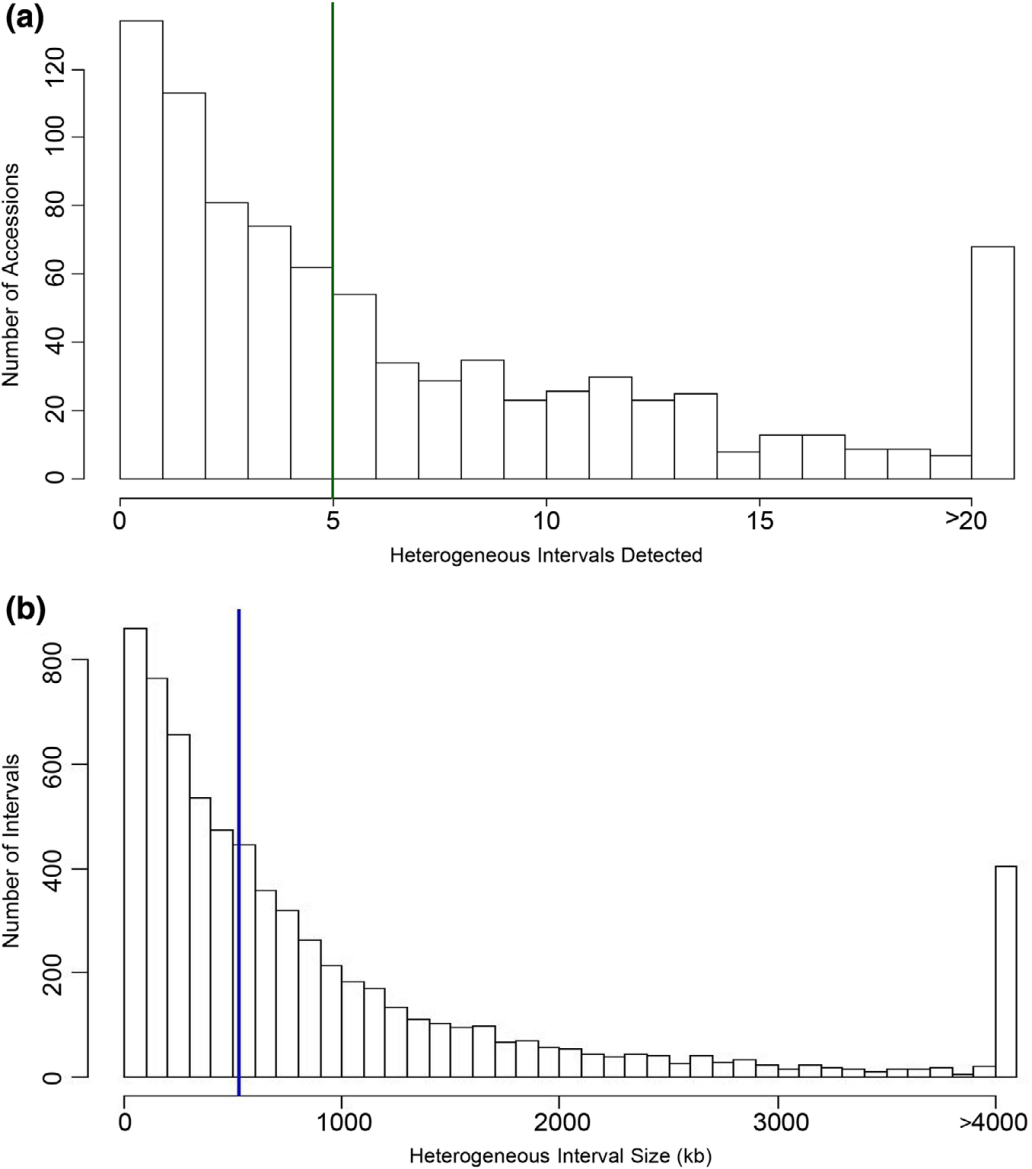
Histograms of heterogeneous interval detection. (a) Distribution of heterogeneous intervals detected per accession. 870 accessions had a total of 6,925 intervals detected. Green line represents the median number of intervals detected per accession of 5. (b) Distribution of detected heterogeneous genomic interval size. Genomic intervals were determined based off base pairs spanning a detected interval using the physical genomic positions associated with the start and stop of the 42,080 SoySNP50K Chip markers. Blue line represents the median genomic interval size of 532 kilobases (kb)

Heterogeneous intervals were assessed among all accessions and within groups of wild (*G. soja*), landrace, North American cultivars, and unclassified *G. max* accessions to explore differences in heterogeneity between germplasm classes. Landrace and North American cultivar classes were defined by Song et al. (2015) upon release of the USDA SGC SoySNP50K genotyping data. Unclassified *G. max* accessions comprise a combination of landraces, North American cultivars, non‐North American cultivars, NILs, mutants, and other unreleased breeding materials that were not previously defined in Song et al. (2015). The rates of heterogeneity from the interval detection were assessed between the 870 *G. soja*, landrace, North American cultivar, and Unclassified *G. max* accessions that were detected out of the 20,087 accessions in the USDA SGC (Table 2). The mean number of heterogeneous intervals detected per accession was eight, while the median was five. Most accessions had very few intervals detected, having a minimum and mode of one interval, though the interval count reached a maximum of 91. Due to these factors, the histogram of intervals detected per accession showed a highly right‐skewed distribution (Figure 2a). The overall mean and median interval size were determined on both a SNP and physical genomic size basis. The size of the interval was determined based on the 42,080 SNPs and their physical positions from the SoySNP50K Chip (see Materials and Methods for details). The two interval size calculations are complementary to one another, as the SNP markers are not equally distributed throughout the genome and do not reflect accurate genomic distance, particularly when comparing heterochromatic and euchromatic regions. The mean and median interval size in SNPs were 65 and 40 SNPs, respectively, showing the interval sizes to be right‐skewed. The mean and median interval sizes in kilobases were 1,298 kb and 532 kb, respectively, also showing a right‐skewed distribution of detected genomic interval sizes (Figure 2b).

**TABLE 2:** Results of the heterogeneous interval detection for 20,087 *G. max* and *G. soja* accessions from the USDA Soybean Germplasm Collection (SGC). For each of four germplasm classes the number of accessions with heterogeneous intervals detected is presented, along with the total number of intervals, and the mean and median number of intervals per accession in that class

Germplasm Class^a^	Total Accessions in SGC	No. of heterogeneous accessions (% SGC)^b^	Total no. of intervals	Mean intervals per accession	Median intervals per accession
*G. soja*	1,178	7 (0.6%)	147	21	21
Landrace	5,396	278 (5.2%)	2,372	8.5	5
North American Cultivar	562	27 (4.8%)	164	5.9	3
Unclassified *G. max*	12,951	558 (4.3%)	4,242	7.6	5
Total	20,087	870 (4.3%)	6,925	8	5

a *G. soja* is an interfertile wild relative of *G. max*. Landrace and North American cultivars were identified from Song et al. (2015). Unclassified *G. max* denotes any *G. max* that were not identified as Landrace or North American cultivar by Song et al. (2015).

b Number of heterogeneous accessions is based on the complete analysis pipeline, including both initial screening and heterogeneous interval detection.

Among the 1,178 *G. soja* accessions in the USDA SGC, seven (0.6%) were identified through the accession scanning and interval detection pipeline (Table 2). This *G. soja* accession detection rate was nearly eight times lower than the *G. max* proportion of 4.6%. The *G. soja* accessions that were detected exhibited more heterogeneous intervals and greater heterogeneous genome coverage, on average, than the *G. max* accessions (Tables 2, 3, 4). However, the mean and median heterogeneous interval size was similar to other germplasm classes (Tables 3, 4). When considering all of the intervals within a given accession, the mean and median coverage of the *G. soja* genome by heterogeneous intervals were both approximately 30 Mb of the roughly 1 Gb genome. These mean and median genomic intervals were approximately three‐ and eight‐fold more base pair coverage than the *G. max* germplasm classes, respectively.

**TABLE 3:** Distribution of SNPs within heterogeneous intervals or accessions identified by the heterogeneity analysis pipeline. Heterogeneous intervals may contain a combination of heterozygous and homozygous SNPs

Germplasm Class^a^	Mean no. SNPs per interval	Median no. SNPs per interval	Mean no. SNPs per accession	Median no. SNPs per accession
*G. soja*	71	40	1,499	1,489
Landrace	60	38	515	273
North American Cultivar	70	53	427	221
Unclassified *G. max*	67	41	510	287
All classes	65	40	517	284

a Germplasm classes of the USDA Soybean Germplasm Collection are based on the work of Song et al. (2015).

**TABLE 4:** Mean and median lengths (kb) of heterogeneous intervals among USDA Soybean Germplasm Collection accessions belonging to four germplasm classes (Song et al., 2015). Interval lengths were based on the physical positions of the first and last heterozygous SNPs that defined an interval, as determined by the heterogeneity analysis pipeline

Germplasm Class	Mean kb per interval	Median kb per interval	Mean kb per accession	Median kb per accession
*G. soja*	1,409	585	29,585	32,111
Landrace	1,159	500	9,891	4,153
North American Cultivar	1,270	555	7,713	2,289
Unclassified *G. max*	1,372	560	10,432	4,904
All classes	1,298	532	10,329	4,519

The *G. max* germplasm classes of landrace, North American cultivar, and Unclassified all had similar proportions of heterogeneous accessions (Table 2). Both landraces and Unclassified *G. max* had similar mean and median numbers of intervals detected, while North American cultivars had slightly lower mean and median intervals detected (Table 2). The North American cultivars also displayed a lower mean and median genome coverage of all intervals in a given accession with respect to both SNP and kilobase size (Tables 3, 4). However, the North American cultivar data is derived from a small sample size, consisting of only 164 intervals from 27 accessions, limiting the utility of this comparison.

### 3.3 Heterogeneous intervals detected per chromosome

**FIGURE 3:**
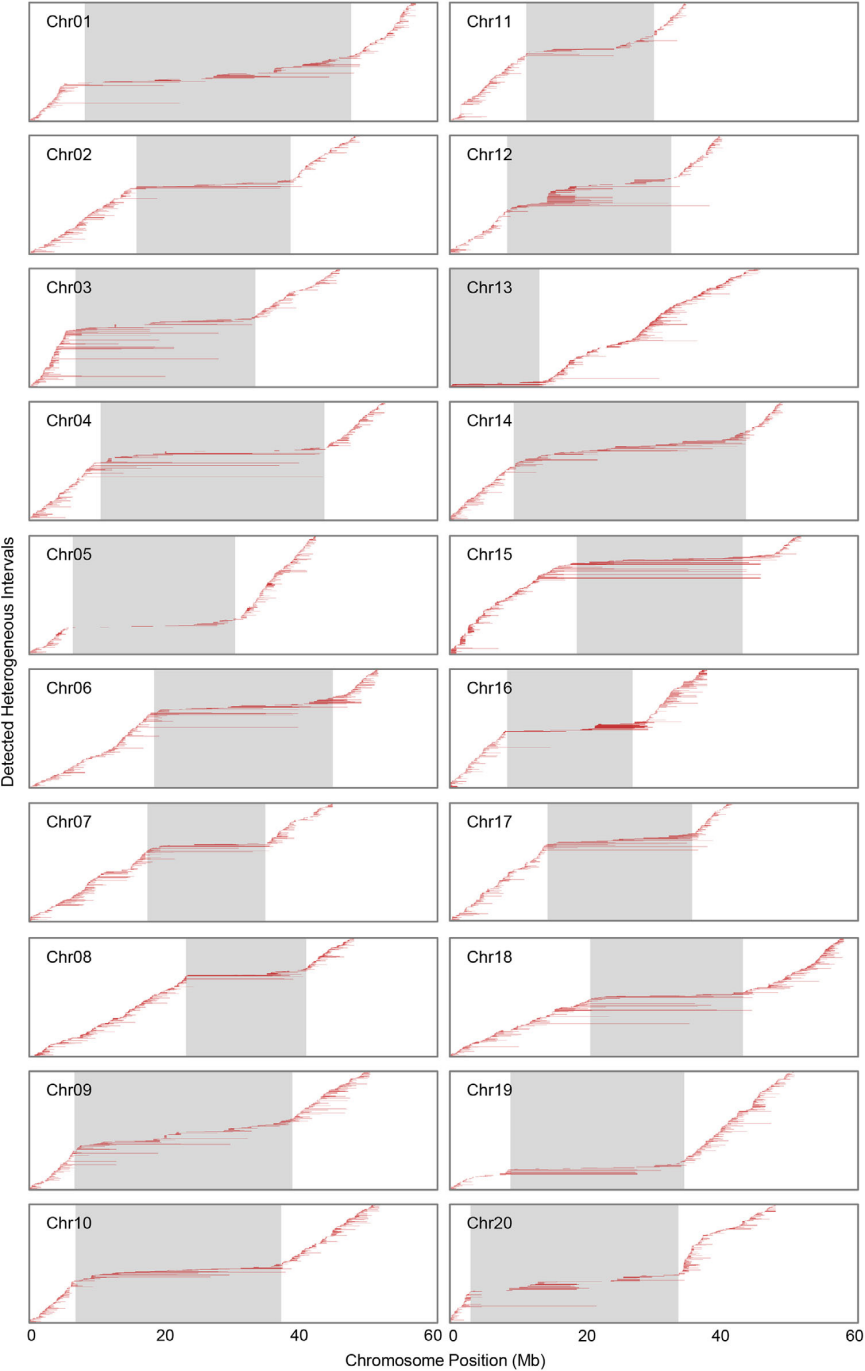
Heterogeneous intervals in the soybean genome. Each panel represents one of the 20 *G. max/soja* chromosomes. Each red bar represents a single interval detected on one accession, with the bars stacked in the ascending order of their physical start position with respect to the *G. max* reference genome ‘Williams 82’ Assembly 2 SoySNP50K Chip positions. White regions denote approximate euchromatic regions, and gray shaded regions denote heterochromatic regions from Song et al. (2016)

**FIGURE 4:**
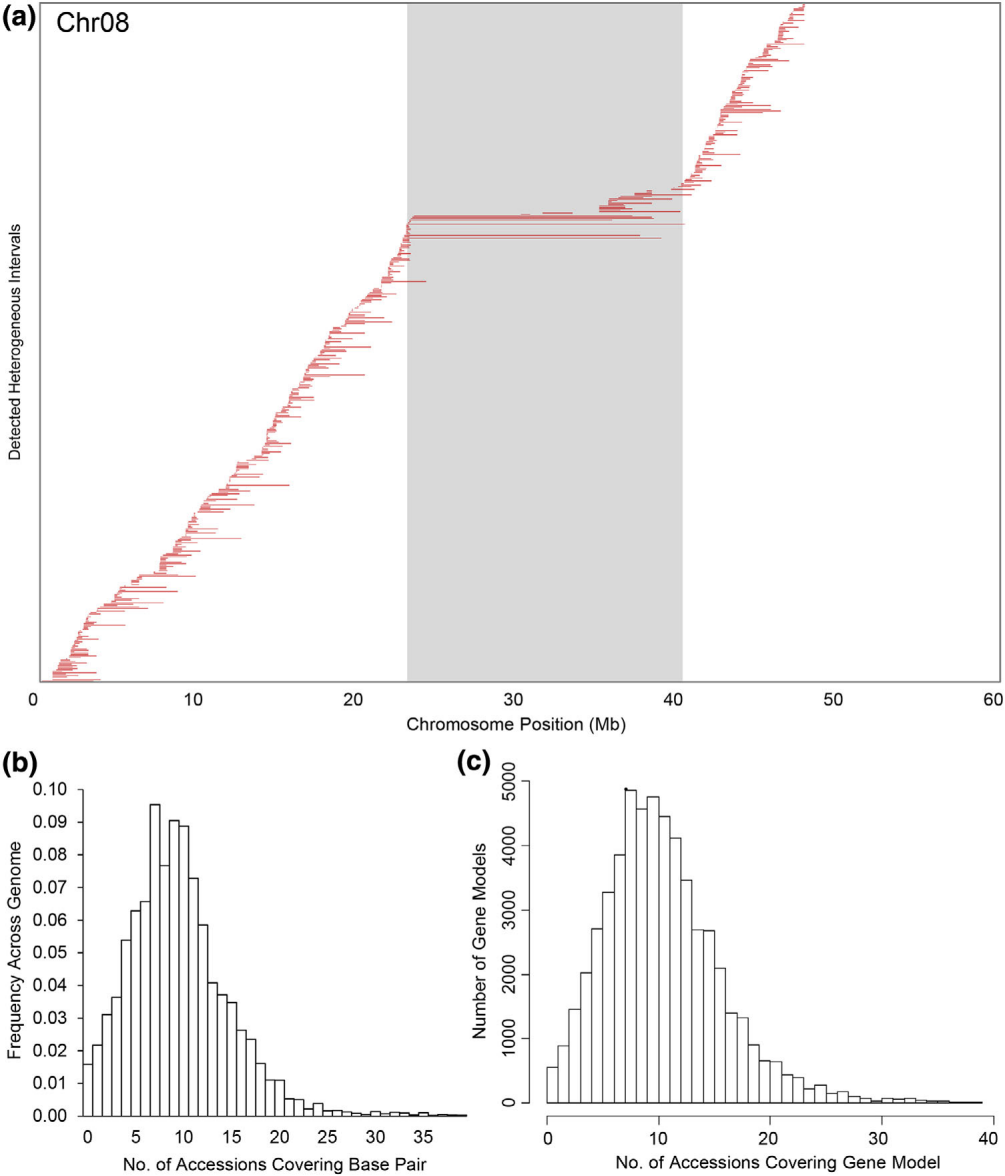
Coverage of detected heterogeneous intervals. Part (a) shows the distribution of detected heterogeneous intervals across chromosome 08. Each red bar represents a single interval detected on one accession, with the bars stacked in the ascending order of their physical start position with respect to the *G. max* reference genome ‘Williams 82’ Assembly 2 SoySNP50K Chip positions. White regions denote approximate euchromatic regions. Gray shaded regions denote heterochromatic regions from Song et al. (2016). Part (b) shows the distribution of the number of heterogeneous accessions per base pair across the soybean genome. Part (c) shows the distribution of the number of heterogeneous accessions per gene model across the soybean genome

Overall, 98.4% of the bases and 99.0% of the gene models in the soybean genome were covered by at least one heterogeneous interval. The physical positions of each of the detected intervals were plotted along the chromosomes using the ‘Williams 82’ Assembly 2 SoySNP50K Chip positions as the reference, stacked in ascending order of their physical start position (Figure 3). The corresponding euchromatic and heterochromatic regions of *G. max* (assembly Wm82.a2) were identified based on Song et al. (2016). A detailed representation of the distribution of heterogeneous intervals detected across chromosome 08 is shown in Figure 4a. This example displays a view of the size, distribution, and depth of heterogeneous intervals among accessions along this chromosome.

The soybean genome consists of approximately one billion base pairs and 55,589 chromosomal gene models. The number of accessions with heterogeneous intervals covering each base pair and gene model are respectively shown as distributions in Figure 4b and 4c. The number of heterogeneous accessions covering each base pair had a mean of 9.5X and a median of 9X per base pair (Figure 4b). The number of heterogeneous accessions covering each gene had a mean of 9.2X and a median of 9X per gene model (Figure 4c). The number of gene models covered by heterogeneous intervals per accession ranged from 0 to 8,369, with a mean of 631 and median of 345 gene models. The heterochromatic regions of the chromosomes were easily distinguishable from the euchromatic regions when examining heterogeneous interval size and frequency. Relatively few intervals were found within heterochromatic regions, however, they tended to cover larger genomic regions (Figures 3 and 4a). Conversely, many intervals were located within the euchromatin, though they tended to be relatively short. The differences in interval size and frequency in the heterochromatin and euchromatin is presumably caused by higher rates of recombination in the euchromatin compared to the heterochromatin, but may also be influenced by the higher SNP marker densities in the euchromatin regions. Euchromatin regions exhibited slightly higher interval coverage, with a mean of 10.0X and median of 9X, compared to a mean of 7.7X and median of 8X in heterochromatin regions. It appears that similar intervals were often represented by multiple accessions. For example, chromosome 08 had seven intervals that all started at position 786,288. Chromosome 11 had the same 7‐SNP interval detected in 25 different accessions from position 1,599,720 to 1,602,125. Moreover, several large gaps in interval coverage occurred across the heterochromatic regions of chromosomes 05 and 20 (Figure 3).

### 3.4 Validation of heterogeneity

Heterogeneous intervals were validated using Sanger sequencing of PCR amplicons for ten different accessions. For each accession, twelve individual plants were used as templates for PCR amplification within genomic intervals that displayed a heterozygous SNP in the SoySNP50K Chip dataset. PCR primer sequences specific to these locations are provided in Supplemental Table S2. Each of the ten selected accessions exhibited exactly one putatively heterogeneous locus in the SoySNP50K Chip data. Furthermore, the ten accessions could be divided into three groups: (1) Accessions that are purposefully maintained as heterozygotes by the USDA SGC for a specific locus, and the heterozygous SoySNP50K calls were located within the known locus; (2) Accessions that are purposefully maintained as heterozygotes by the USDA SGC for a specific locus, but the heterozygous SoySNP50K calls were located at a different locus; (3) Accessions that are not maintained as heterozygotes by the USDA SGC, but exhibited a single heterozygous locus based on the SoySNP50K calls. Regardless of category, all ten loci/accessions validated as heterogeneous within each family (Table 5). Eight of the families exhibited a heterogeneous mixture of homozygous genotypes. Two accessions (PI 548203 and PI 548245) exhibited a heterogeneous mixture of both heterozygous and homozygous genotypes. These results fit expectations, as those two accessions are maintained by the USDA SGC as heterozygotes for sterility or lethal alleles at loci that fell within the heterogeneous intervals detected in our study.

**TABLE 5:** Validation of heterogeneity among a sample of ten accessions in which a single heterogeneous region was detected. Six accessions are purposefully maintained as heterozygous at sterility or lethality loci, and the heterogeneous region detected in two of these encompassed the expected locus. Primers were designed to amplify a region surrounding a putative heterogeneous SNP, and alleles from the amplified product were confirmed via Sanger sequencing. The observed alleles for each SNP and their ratio among 11 to 12 plants from each accession is also reported

Accession	Line Notes and Maintenance^a^	Target SNP^b^	Chromosome	Expected Nature of Heterogeneity at Target SNP^c^	Observed Genotypes	Ratio	Interpretation
PI 79712	landrace, standard maintenance	ss715607317	Gm10	heterogeneous, homozygous	[AA]:[GG]	4:8	Confirmed heterogeneity at expected locus
PI 423780B	landrace, standard maintenance	ss715635924	Gm19	heterogeneous, homozygous	[AA]:[GG]	3:8	Confirmed heterogeneity at expected locus
PI 542043	cultivar, standard maintenance	ss715602284	Gm08	heterogeneous, homozygous	[AA]:[GG]	7:5	Confirmed heterogeneity at expected locus
PI 595926	cultivar, standard maintenance	ss715621959	Gm15	heterogeneous, homozygous	[AA]:[GG]	2:9	Confirmed heterogeneity at expected locus
PI 548237	experimental line with Ms1 male sterility, maintained as heterozygous	ss715627934	Gm17	heterogeneous, homozygous	[CC]:[AA]	9:3	Confirmed heterogeneity at expected locus
PI 548247	experimental line with y22 associated chlorosis, maintained as heterozygous	ss715588194	Gm04	heterogeneous, homozygous	[TT]:[GG]	1:11	Confirmed heterogeneity at expected locus
PI 548264	experimental line with Ms1 male sterility, maintained as heterozygous	ss715598205	Gm07	heterogeneous, homozygous	[AA]:[GG]	4:7	Confirmed heterogeneity at expected locus
PI 548267	experimental line with Ms1 male sterility, maintained as heterozygous	ss715581305	Gm02	heterogeneous, homozygous	[AA]:[GG]	6:5	Confirmed heterogeneity at expected locus
PI 548203	experimental line with y22 lethal chlorosis, maintained as heterozygous	ss715615554	Gm13	heterogeneous, homozygous and heterozygous	[CC]:[CT]:[TT]	4:6:2	Confirmed heterogeneity at expected locus
PI 548245	experimental line with Ms1 male sterility, maintained as heterozygous	ss715614096	Gm13	heterogeneous, homozygous and heterozygous	[GG]:[GT]:[TT]	3:5:4	Confirmed heterogeneity at expected locus

a Based on data available from the USDA Germplasm Resources Information Network (GRIN). Accessions with known sterility or lethal alleles are maintained as heterozygous.

b Within the detected region of putative heterogeneity for each accession a single heterogeneous SNP was selected for amplification.

c Accessions were expected to be either a heterogeneous mixture of homozygous individuals, or a heterogeneous mixture of homozygous and heterozygous individuals.

## 4 DISCUSSION

### 4.1 Strengths and limitations of heterogeneity detection of the USDA SGC using the SoySNP50K chip

The goal of this study was to identify genetic heterogeneity within accessions of the USDA SGC. While the presence of genetic heterogeneity persisting within highly homogeneous and inbred soybean has been previously demonstrated (e.g., Haun et al., 2011), the recent genotyping of all 20,087 accessions of *G. soja* and *G. max* in the USDA SGC has facilitated a more high‐throughput and expansive analysis in this study. Our analysis pipeline revealed putative heterogeneity in 870 accessions, with the majority of these accessions exhibiting more than three heterogeneous chromosomes or intervals (Table 1; Figure 2a).

The analysis from this study provides first glimpse of within‐accession heterogeneity in the soybean germplasm, however there are several factors that limit the ability to detect and resolve this phenomenon in greater depth. Notably, this study was predicated on the concept that multiple individuals (typically three) of an accession were pooled for the USDA SGC SoySNP50K Chip genotyping. Three individuals compose a relatively small sample and much of the true heterogeneity within an accession may have gone unsampled. Furthermore, it is unclear whether the pooling method was entirely consistent for all accessions. For instance, even though within‐accession variation was previously found in ‘Williams 82’ (Haun et al., 2011), the ‘Williams 82’ in the USDA SGC (PI 518671) analyzed in this study did not have did not pass the heterogeneous accession screening step. There are at least three explanations as to why this ‘Williams 82’ sample did not exhibit heterogeneity: (1) the ‘Williams 82’ stock at the USDA SGC may have been bottlenecked to a single or few individuals at one point in time and it is completely homogenous among all individuals, differing from other stocks; (2) the approximately three bulked individuals used for SoySNP50K genotyping may have coincidently had identical genotypes, while individuals with heterogeneity were simply not sampled; (3) the ‘Williams 82’ sample in the SoySNP50K genotyping may have been derived from a single individual rather than a pooled sample. Regardless of the cause, the lack of heterogeneity of the ‘Williams 82’ sample in the SoySNP50K analysis suggests that similar processes may have also masked true heterogeneity in other accessions as well. Further support for this statement can be observed in the PCR validation experiments in this study. While all ten accessions validated as heterogeneous, in some cases the ratio of genotypes was highly skewed (e.g, 1:11 for PI 548247). Thus, the ability to detect heterogeneity within an accession is a direct function of the likelihood that polymorphic individuals will be present in a sampling of approximately three individuals. Clearly, heterogeneous loci with skewed allelic ratios among the individuals in the accession are less likely to be detected with shallow sampling.

Furthermore, the SoySNP50K Chip has a finite ability to resolve polymorphic haplotypes. The Chip density is 1 SNP every 9.1 kb in euchromatic regions and 1 SNP every 49.1 kb in heterochromatic regions, and was organized to evenly distribute these SNPs within both types of regions. While this is a high density, smaller heterogeneous intervals are not as likely to be detected as large intervals. Only about 3% of the heterogeneous intervals detected were under 27.3 kb in length (i.e., the minimum of three SNPs required for detecting an interval multiplied by the average euchromatic SNP density of 9.1 kb). The distribution of these markers could also be truncating heterogeneous intervals that extend past their detected SNP boundaries but end prior to the adjacent SNP. Furthermore, the SoySNP50K Chip also does not capture the entire length of each chromosome, so some gene models nearest to the beginnings and ends of chromosomes fall outside of the regions that were scanned for heterogeneous intervals.

Given the factors described above, one can assume that within‐accession heterogeneity is more common in the USDA SGC collection than is reported in this study. Researchers should be aware that (i) some accessions that did not exhibit heterogeneity in this study do indeed have heterogeneity present in the seed stock, and (ii) there may be many additional within‐accession heterogeneous loci that were not detected by this study due to the limited sample size used for genotyping.

### 4.2 Proportions of heterogeneity in different germplasm classes

The soybean germplasm used in this study was diverse, containing 20,087 accessions that included the *G. soja*, landraces, elite cultivars, as well as many other research and breeding materials. While the collection does not clearly define many of these germplasm classes, Song et al. (2015) was able to define a subset of germplasm as “wild”, “landrace”, and “North American cultivar” to evaluate features such as genetic relationships, geographic origin, linkage disequilibrium, and genomic structure. We used this subset in our study to compare heterogeneous intervals from different germplasm classes. Since breeding efforts purposefully make crosses between genetically diverse germplasm, we predicted that more North American cultivars would have a higher proportion of detected heterogeneity than landraces, and that a higher proportion of landraces would have detected heterogeneity than *G. soja* accessions.

The proportion of accessions with heterogeneity detected in *G. soja* was low compared to landraces and North American cultivars. This is not surprising since *G. soja* has not been subjected to man‐made manipulation. Like cultivated soybean, *G. soja* is primarily a self‐pollinating species, so within‐accession homogeneity could result from the collection of progeny of one homozygous plant or multiple homogenous plants (Supplemental Figure S2A). Alternatively, a *G*. soja accession could be collected from diverse plants which would result in widespread heterozygous SoySNP50K Chip calls that would be filtered out of our pipeline (Supplemental Figure S2B). Though infrequent, outcrossing is known to occur within soybean and may explain the few heterogeneous *G. soja* accessions observed here (Fujita, Ohara, Okazaki, & Shimamoto, 1997).

We initially expected that North American cultivars would have the highest proportion of heterogeneity, driven by modern breeding strategies such as backcrossing and recurrent selection. For example, Haun et al. (2011) showed that heterogeneity in ‘Williams 82’ was caused by incomplete homogeny of the cultivar upon bulk‐harvesting, primarily in the site where Phytopthora root rot resistance from ‘Kingwa’ was introgressed into ‘Williams’ to the BC6F3 generation. Other breeding methods could result in similar heterogeneity, such as recurrent selection or single seed decent where lineages are bulked before becoming fully homozygous. However, the heterogeneous interval detection analysis we employed showed a similar proportion of accessions with heterogeneity in landraces and North American cultivars. It is unclear why the proportion of accessions with heterogeneity was similar in these two groups, as the North American cultivars would presumably have undergone a more recent and intense process of artificial selection to introduce heterogeneity. However, the loose categorization of landraces creates some uncertainty, as it encompasses a wide spectrum of accessions that are cultivated *G. max* but have not been substantially altered by modern breeding methods. Like North American cultivars, landraces have been subjected to some degree of purposeful selection from the time of domestication through the nineteenth century. Conversely, like *G. soja*, outcrossing likely occurs occasionally in landrace accessions. Moreover, the mode of initial collection for many landrace accessions is unknown, but likely variable. Thus, it is interesting that the proportions of heterogeneous accessions detected from landrace and North American cultivar classes are similar even though the processes that created them differ.

### 4.3 Heterogeneity detection as a population‐level reverse genetics resource

Near isogenic lines (NILs) are frequently created to generate small targeted regions of genetic diversity which enable the identification causal loci. In soybeans, an extensive isoline collection exists (Bernard, Nelson, & Cremeens, 1991) with phenotypic differences for a variety of traits and molecular mapping resources (Muehlbauer et al., 1991; Muehlbauer, Specht, Staswick, & Graef, 1989). The NILs from the USDA Soybean Isoline Collection have been successfully used in a variety of applications, such as genetic fine‐mapping (Severin et al., 2010), genomic physical/structural mapping (Stec et al., 2013), and validating trait‐loci associations (Peiffer et al., 2012). While NILs are useful for trait mapping and characterization, their creation is costly and time‐consuming, involving bi‐parental crosses and backcrossing to introgress and map a trait of interest.

**FIGURE 5:**
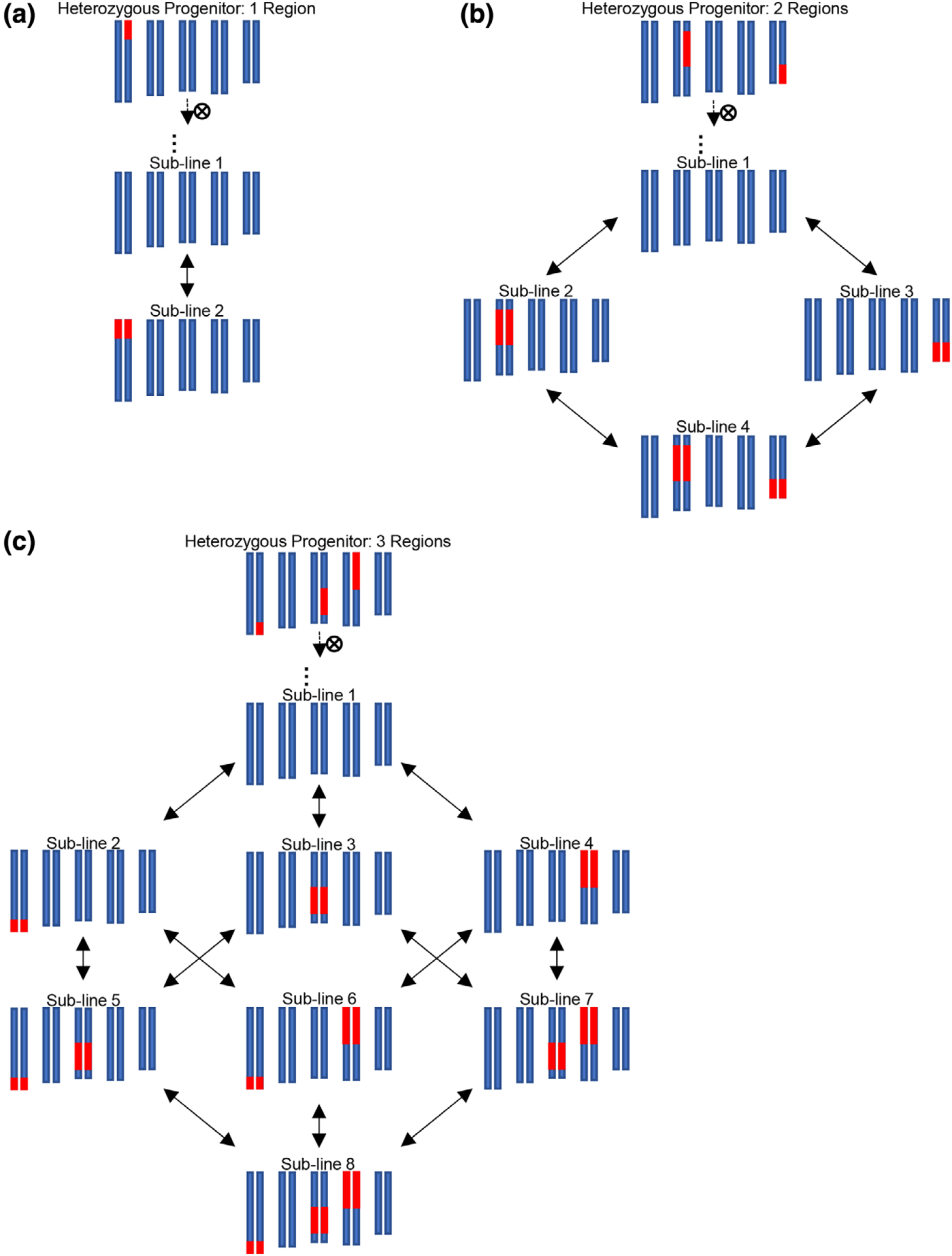
Examples of near isogenic sub‐line generation from USDA SGC accessions. Individuals from accessions with heterogeneity originated from a heterozygous progenitor that was selfed and bulk harvested repeatedly, resulting in sub‐lines within a single accession. Sub‐lines that are near‐isogenic can be identified as pairs and used as near‐isogenic lines (NILs). Example outcomes of sub‐lines are shown for (a) one, (b) two, and (c) three heterogeneous regions if no recombination occurs. As the number of heterogeneous regions increases, the number of potential sub‐line genotypes increases exponentially. Bars represent soybean chromosomes, with blue denoting regions of the predominant haplotype, and red denoting regions of an alternative haplotype. The dashed arrows, crosses, and ellipses represent repeated selfing and bulk harvesting. Each solid double‐headed arrow represents one NIL pair

As described in this study, heterogeneous accessions in the USDA SGC collection could be used for rapid NIL development for virtually any region of the genome. The SoySNP50K data reflects the genotype of the heterozygous progenitor and projects a composite of the existing sub‐lines in the accession (Figure 5). The heterogeneous accessions detected in our analysis, in most cases, are expected to have multiple sub‐lines per an accession. As the number of heterogeneous regions increases, the number of potential sub‐line genotypes increases exponentially, as does the number of potential NIL pairs. Genotyping of multiple individuals in an accession can potential identify numerous pairs that respectively represent NILs for different regions of the genome (Figure 5). While it may be advantageous to have many different NIL pair possibilities, the higher numbers of regions and resulting sub‐lines and pairs increases the complexity and labor involved in the family segregation and characterization. This approach is loosely based on Tuinstra, Ejeta, and Goldsbrough (1997) who identified residual heterogeneity within individual recombinant inbred lines (RILs). These families, termed heterogeneous inbred families (HIFs), can then be genotyped to identify NILs. Our strategy, alternatively, identifies already‐existing heterogeneity among individuals within standing accessions, as opposed to generating heterogeneity *de novo* from bi‐parental population development.

While heterogeneous intervals detected from the USDA SGC could potentially be used as a tool to map traits and clone genes underlying important phenotypes, certain traits and/or genome locations may be more recalcitrant to this approach. The USDA SGC actively works to maintain a pureline collection and would likely cull out individuals with morphologies inconsistent with the predominant phenotype in an accession. Thus, morphological traits that can be easily observed visually (e.g., trichome color, leaf shape, pod size) are less likely to be maintained as heterogeneous within a given USDA SGC accession. This resource may be more applicable for morphologically subtle or carefully‐measured traits, such as seed composition, branch angle, or abiotic stress tolerance.

## 5 CONCLUSIONS

This is the first study to examine and characterize within‐accession heterogeneity in an extensive collection of soybean. After analyzing over 20,000 wild and cultivated soybean accessions, we identified 6,925 intervals of heterogeneity within 870 soybean accessions maintained by the USDA SGC. These heterogeneous loci covered 99% of the gene models in the soybean genome and represent an extensive genetic resource that is immediately available for both forward and reverse genetics studies. This study provides a valuable window into soybean genetic heterogeneity and its crop improvement potential.

## CONFLICT OF INTEREST

The authors declare that there is no conflict of interest.

## Supporting information

Supplemental Table S1. List of all intervals detected by the heterogeneity analysis pipeline.

Supplemental Table S2. Primers designed to amplify ten target SNPs within heterogeneous intervals detected by the analysis pipeline.

Supplemental Figure S1. Concept for heterogeneity detection.
Supplemental Figure S2. Examples of heterogeneity patterns.
