# Supplementary material for: Characterization of genetic heterogeneity within accessions in the USDA soybean germplasm collection
Source: Plant Genome. 2020 Apr 6;13(1):e20000. doi: 10.1002/tpg2.20000 (PMC12807376; doi:10.1002/tpg2.20000)
Supplement: Supplementary file 1 [file TPG2-13-e20000-s001.pdf]

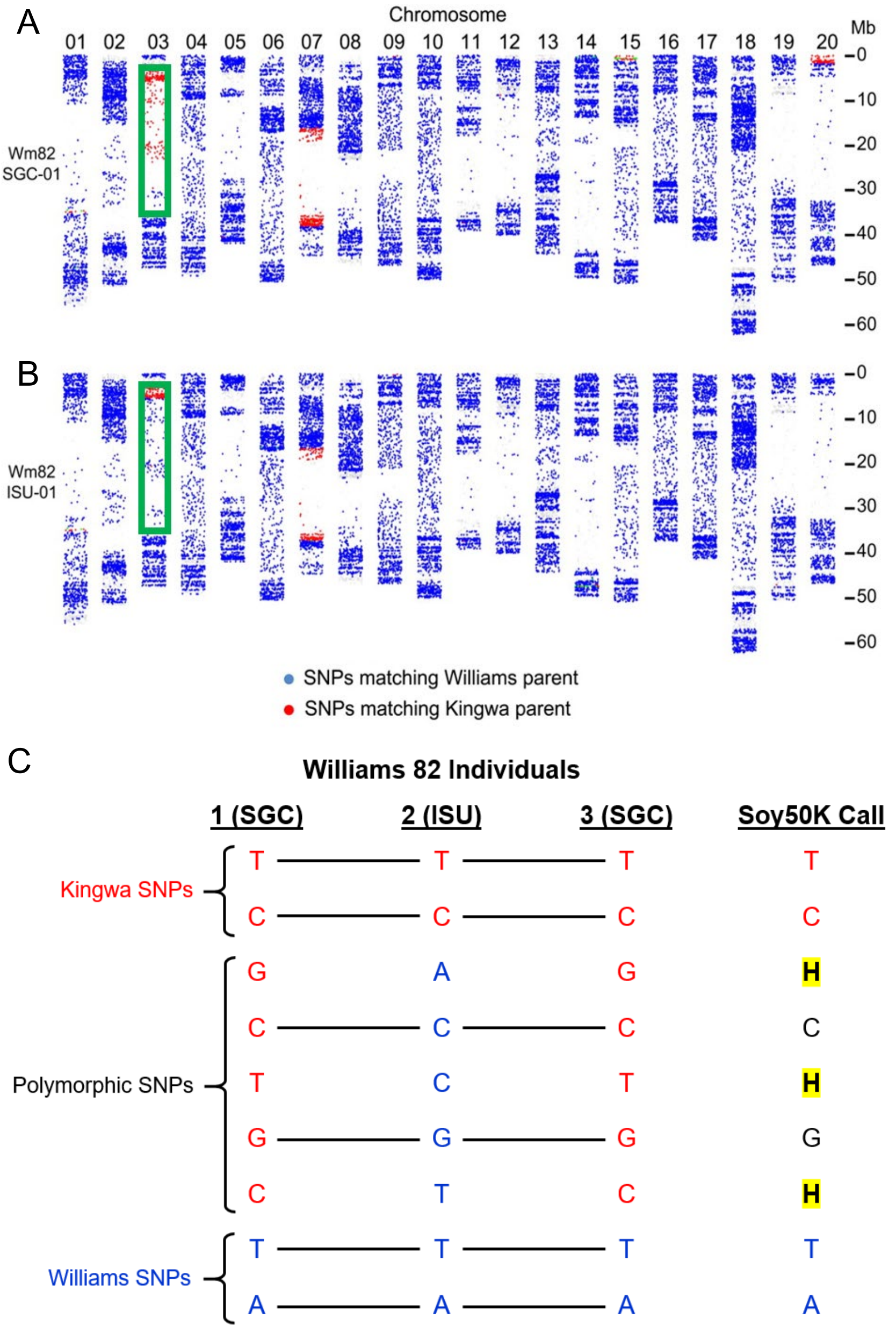

Supplemental Figure S1. Concept for heterogeneity detection. Intracultivar variation was demonstrated in the soybean reference cultivar, 'Williams 82' (Wm82), by Haun et al. (2011) through SNP genotyping of (A) an individual maintained at the USDA-ARS Soybean Germplasm Collection (SGC) and (B) an individual maintained at Iowa State University (ISU). Note: (A) and (B) were adapted from Haun et al. (2011). Blue dots represent SNP positions from the 'Williams' parent genotype, while red dots indicate SNP positions from the 'Kingwa' parent genotype. The green boxes indicate a corresponding interval on chromosome 3, which is represented and simplified in the theoretical example in (C). If a mixture of plant material from 3 individuals (in this case, two SGC and one ISU) were pooled and genotyped on the SoySNP50K Chip, SNP calls that differ across SGC and ISU will be polymorphic and thus will show up as clusters of heterozygous calls. Clustered heterozygous SNP calls can therefore be used to indicate heterogeneous regions within an accession.

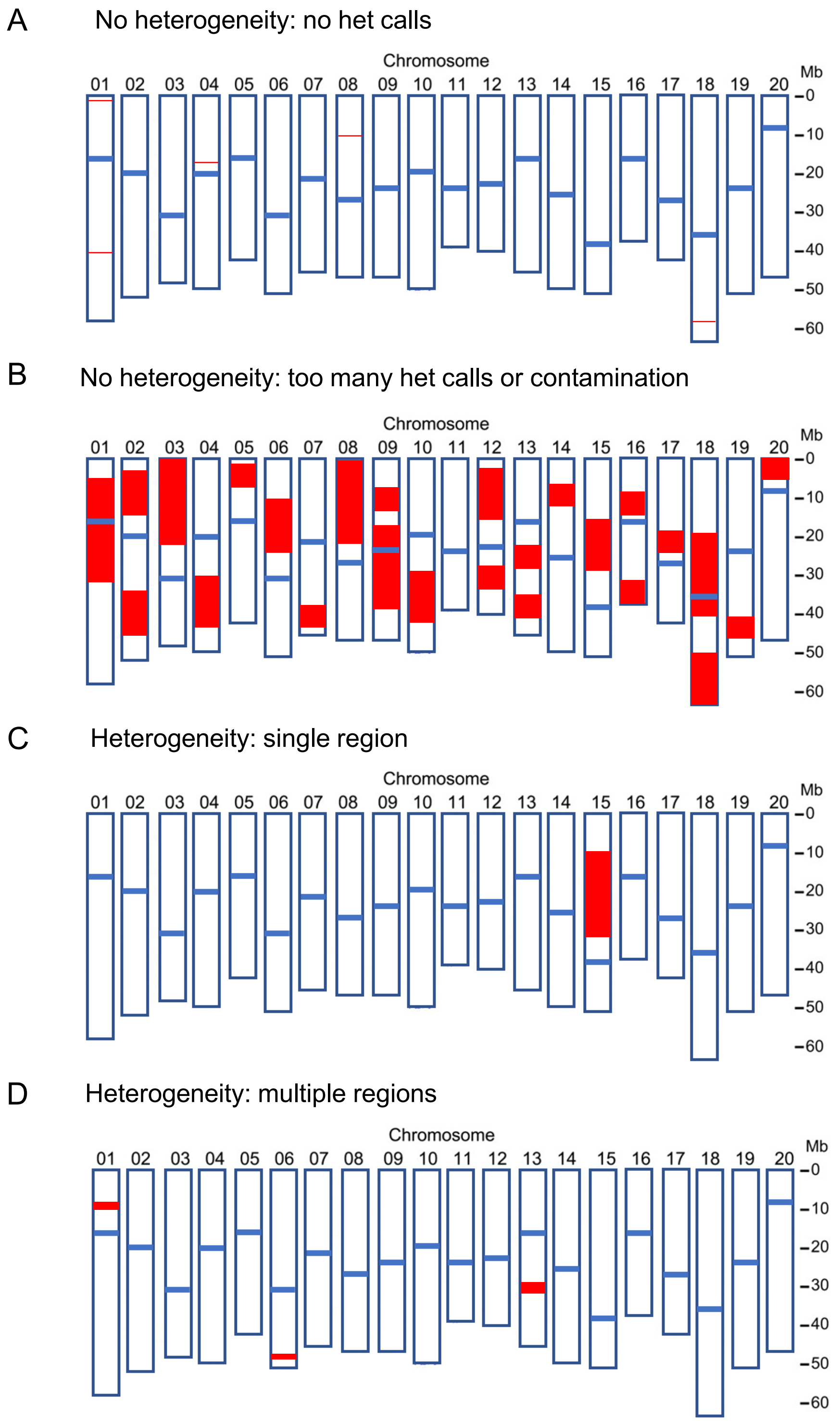

Supplemental Figure S2. Examples of heterogeneity patterns. White bars outlined in blue represent each chromosome of the soybean genome with approximate length in megabases (Mb). Horizontal blue bars within each chromosome represent approximate centromeric regions. Red bars indicate occurrences or clusters of heterozygous calls made in the SoySNP50K data (Song et al., 2015) for a given accession. (A) Example of an accession detected as not heterogeneous due to low heterozygous call rates. (B) Example of an accession detected as not heterogeneous due to no disproportionate chromosomal distribution of heterozygous calls. (C) Example of an accession detected with one region of heterogeneity. (D) Example of an accession detected with multiple regions of heterogeneity.
